# Pulmonary nocardiosis with hilar mass misdiagnosed as lung cancer: A case report

**DOI:** 10.1097/MD.0000000000042524

**Published:** 2025-05-16

**Authors:** Qian Liu, Qian Li, Bing Liu, Tian Fu

**Affiliations:** a Department of Pulmonary and Critical Care Medicine, Jining First People’s Hospital, Jining, Shandong Province, China.

**Keywords:** lung cancer, metagenomic next-generation sequencing, misdiagnosis, pulmonary nocardiosis

## Abstract

**Rationale:**

Pulmonary nocardiosis (PN) is an opportunistic infectious disease caused by *Nocardia* of the lungs, which lacks specificity in clinical symptoms and imaging, is rare in individuals with normal immune function, and is highly prone to clinical misdiagnosis and missed diagnosis.

**Patient concerns:**

A 55-year-old woman with normal immune function was admitted to the hospital due to a cough and expectoration for more than 20 days.

**Diagnoses:**

Chest computed tomography revealed a mass in the left hilum and obstructive atelectasis in the lingular segment of the left upper lobe. Initially suspected to be a malignant tumor, bronchoscopy with pathological examination suggested inflammation. Finally, metagenomic next-generation sequencing of pathological tissue confirmed PN.

**Interventions:**

After discharge, the patient regularly took trimethoprim-sulfamethoxazole for anti-infective treatment.

**Outcomes:**

Regular follow-ups with the patient revealed that subsequent chest computed tomography scans showed a gradual reduction in the extent of the lesions, and the patient demonstrated a good clinical response and radiological improvement.

**Lessons:**

This case highlights the diagnostic complexity of PN. The radiological manifestations are diverse, commonly including pulmonary consolidation, nodules, and cavities, making it difficult to differentiate from other diseases. Clinicians need to be vigilant and find appropriate testing methods to improve the diagnostic rate of PN.

## 1. Introduction

*Nocardia* is an aerobic, gram-positive, weakly acid-fast staining-positive bacterium. It is ubiquitous in water, air, soil, decaying vegetation, and decomposing organic matter. *Nocardia* mainly enters the human body through inhalation or contact transmission, it is an opportunistic pathogen in individuals with chronic lung disease and in immunocompromised hosts.^[1]^ Pulmonary *nocardiosis* (PN) is a pyogenic or granulomatous lung disease caused by *Nocardia* infection. The majority of *Nocardia* infections affect individuals with compromised immune function, often accompanied by abnormalities in cell-mediated immunity. However, some *Nocardia* species can also infect immunocompetent individuals.^[2]^ *Nocardia* infections can affect various organs, including the brain, lung, skin, and eyes, with the lung being the most commonly involved organ, accounting for approximately 40% to 70% of all *Nocardia* infection cases.^[3]^ The clinical symptoms of PN are nonspecific and include fever, cough, chest pain, dyspnea, anorexia, or weight loss. Radiological imaging can be difficult to distinguish from those of common bacterial infections, invasive pulmonary fungal diseases, tumors, and tuberculosis, the disease may reveal consolidation, nodules, or solitary or multiple lung masses with or without cavitation, potentially leading to misdiagnosis and mistreatment.^[4]^ Currently, our department is treating a patient with PN who presented with no specific clinical symptoms but had imaging findings suggestive of a pulmonary mass. This article reviews the literature to summarize the pathogenesis, clinical imaging manifestations, and treatment of PN, with the aim of providing a reference for early diagnosis and treatment in clinical practice.

## 2. Case report

**Figure 1.**
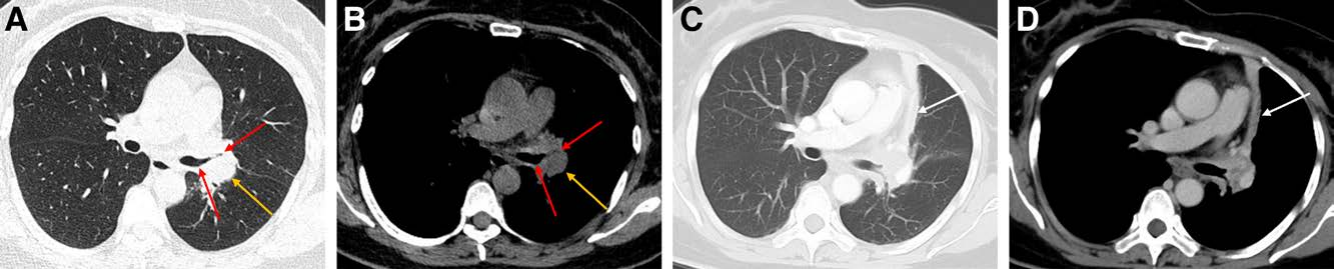
Pretreatment chest CT findings in patient. A mass in the left hilum (yellow arrows) and luminal narrowing of the left main bronchus and left upper/lower lobe bronchi (red arrows). Strip-like consolidative atelectasis in the lingular segment of the left upper lobe (white arrows). CT = computed tomograph.

**Figure 2.**
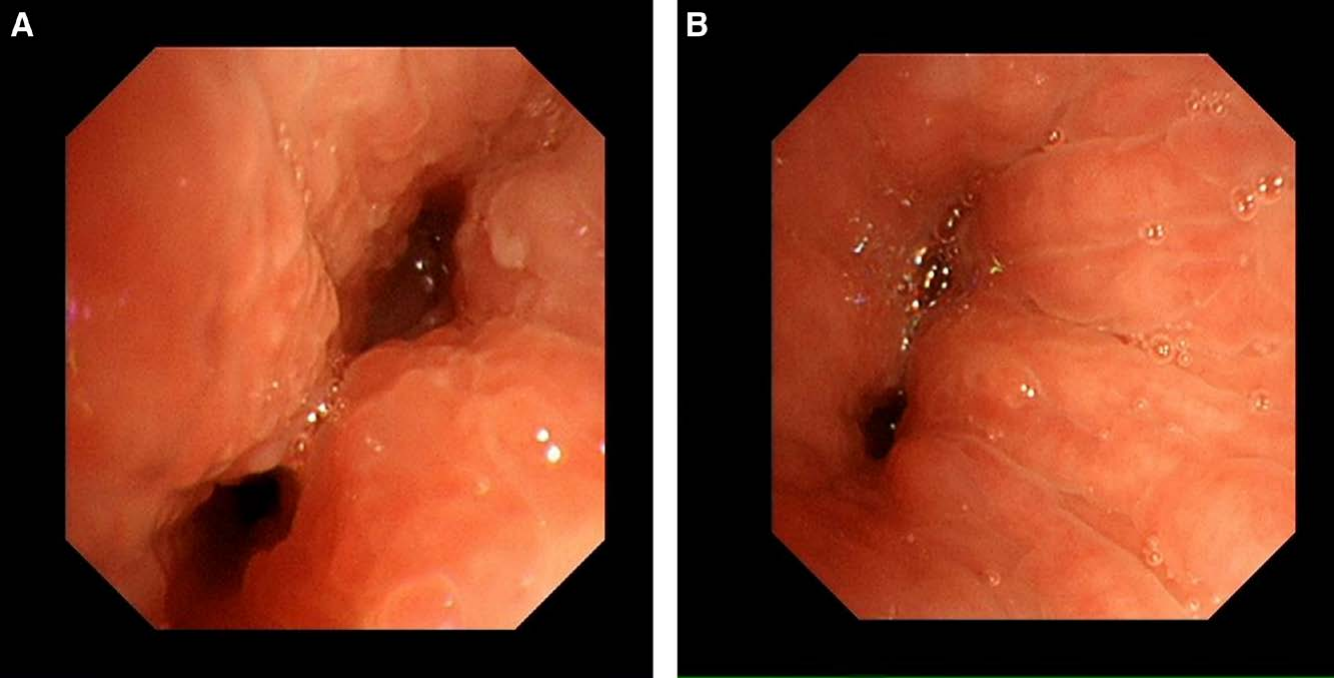
(A, B) Bronchoscopy reveals infiltrative changes in the mucosa at the end of the left main bronchus, and the lumen being almost completely narrowed.

**Figure 3.**
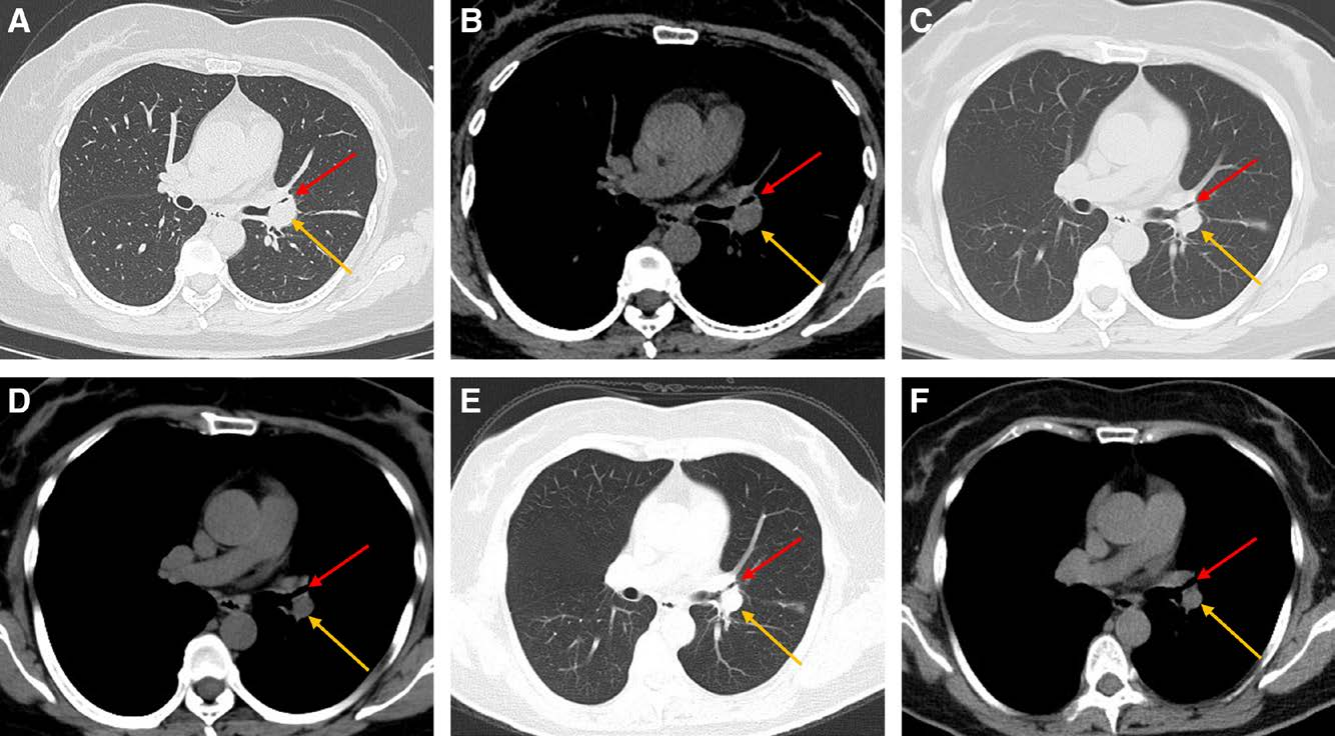
Chest CT findings after treatment with TMP-SMX in patient. (A, B) June 1, 2023, chest CT showed a significant reduction in lesion extent (yellow arrows), and bronchial lumen patency has slightly improved (red arrows). (C, D) Chest CT scan on November 2, 2023. (E, F) Chest CT scan on April 14, 2024. CT = computed tomography, TMP-SMX = trimethoprim-sulfamethoxazole.

A 55-year-old middle-aged woman was admitted to the hospital on April 22, 2023, with a history of cough and expectoration for over 20 days. Approximately 20 days prior, the patient experienced an unexplained onset of cough, producing a small amount of white sputum accompanied by chest tightness and dyspnea, which were predominant with exertion. There were no complaints of chest pain or hemoptysis. Initial antibiotic treatment at a local clinic was ineffective, prompting a visit to our outpatient department. A chest computed tomography (CT) scan revealed an irregular soft tissue density shadow at the left hilar region, with narrowing of the left main bronchus and the bronchi of the upper and lower lobes of the left lung. Additionally, a segmental consolidation shadow was observed in the lingual segment of the left upper lobe (Fig. 1). The patient had a history of partial thyroidectomy and denied any history of hypertension, coronary artery disease, diabetes, or other underlying conditions. There was no history of hepatitis B, tuberculosis, human immunodeficiency virus, or other infectious diseases. Upon admission, the patient’s vital signs were normal. The patient was alert and in good spirits. Auscultation of the lungs revealed coarse breath sounds without adventitious sounds or pleural friction rub. The heart rhythm was regular, and no pathological murmurs were heard in any of the valve areas. The abdomen was soft, without tenderness or rebound tenderness, and there was no edema in the lower extremities. Further laboratory tests showed a C-reactive protein level of 14.48 mg/L, albumin 37.5 g/L, fasting blood glucose 7.76 mmol/L, with no significant abnormalities detected in the complete blood count, liver function, renal function, electrolytes, or coagulation profile. The test for *Cryptococcus neoformans* capsular antigen in blood was negative, suggesting a low likelihood of cryptococcal infection. Additionally, the *Mycobacterium tuberculosis*-specific T-cell detection test yielded negative results, indicating the absence of *M tuberculosis*-specific effector T lymphocytes in the patient’s blood specimen and making pulmonary tuberculosis less probable. A comprehensive ultrasound evaluation of the cervical and supraclavicular lymph nodes demonstrated multiple hypoechoic nodules in the left cervical region and left supraclavicular fossa. These nodules exhibited well-defined margins, rounded morphology (long-axis/short-axis ratio < 2) and loss of fatty hilum. The sonographic features are indicative of lymph nodes with atypical structures, and ultrasound-guided core needle biopsy is recommended for definitive histopathological characterization. Given the patient’s poor response to anti-infective treatment, the presence of atypical structural lymph nodes, and the characteristics of hilar mass on CT imaging, the possibility of lung malignancy was considered high. On April 25th, a contrast enhanced CT scan of the chest was performed, showing no absorption changes in the lesion, with heterogeneous enhancement, and strip-like consolidative atelectasis in the lingular segment of the left upper lobe, suggesting possible lung cancer (Fig. 1). Further bronchoscopy revealed mucosal infiltrative changes at the distal end of the left main bronchus, with the lumen almost completely narrowed, preventing the bronchoscope from entering the upper and lower lobes of the left lung (Fig. 2). Bronchoscopy also favored a diagnosis of lung cancer. Pathological results on April 29th indicated acute and chronic bronchial inflammation with inflammatory and fibrinous exudates. The histopathological nature of the patient’s tissue was difficult to definitively characterize. Immunohistochemical studies were supplemented to assist in diagnosis, with leukocyte common antigen positivity localized to lymphocytes, low molecular weight cytokeratin and cytokeratin positivity localized to epithelial cells, favoring an inflammatory process. The final pathological diagnosis confirmed acute and chronic inflammation of the bronchial mucosa, accompanied by inflammatory infiltrates and fibrinous exudates. Pathologists at Shandong Tumor Hospital reviewed slides and described acute and chronic bronchial inflammation with squamous metaplasia and exudative tissue. Pathology suggested inflammatory disease, and the patient received azithromycin and ceftriaxone for infection during hospitalization but showed little improvement in symptoms. To clarify the nature of the pulmonary lesion, the pathological tissue wax block underwent metagenomic next-generation sequencing (NGS) and detected *Nocardia farcinica* (607 sequences), Supplemental Digital Content, https://links.lww.com/MD/O946. The final diagnosis was PN (*N farcinica*). After discharge, the patient was prescribed oral trimethoprim-sulfamethoxazole (TMP-SMX) at a dose of 2 tablets (400 mg sulfamethoxazole/160 mg trimethoprim) 4 times daily for a 6-month treatment course. Follow-up outpatient chest CT scans on June 1, 2023 and November 2, 2023 showed a significant reduction in lesion extent and bronchial lumen patency has slightly improved (Fig. 3). Another scan on April 14, 2024 (Fig. 3) showed roughly similar lesions to the previous scans.

## 3. Discussion

*Nocardia* was 1st proposed by French veterinarian and microbiologist Edmond Nocard, it commonly invades the human body through the respiratory, digestive, or skin systems, leading to localized or systemic disseminated suppurative inflammation.^[5]^ The Centers for Disease Control and Prevention estimates that there are 500 to 1000 new cases of *Nocardia* infection annually in the United States.^[6]^ The incidence of Nocardiosis is age-specific, most common among elderly patients; the average age of nocardiosis patients is 61.6 years, with 44.0% of patients aged ≥ 65 years and 9.3% < 45 years. The male-to-female ratio is approximately 1.44:1.^[7]^ Among the 119 named species of *Nocardia*, 54 are associated with human infections, and the number of officially published and named subspecies of *Nocardia* has reached 120. The 6 most common species complexes are the *Nocardia nova* complex, the *Nocardia cyriacigeorgica* complex, the *N farcinica* complex, the *Nocardia brasiliensis* complex, the *Nocardia abscessus* complex, and the *Nocardia transvalensis* complex.^[8,9]^ The pathogenic mechanism of *Nocardia* is not yet fully understood, but some studies suggest that its pathogenicity is related to its ability to prevent phagocytosis, specifically by inhibiting the fusion of phagosomes and lysosomes to block the bactericidal action of phagocytic cells, by blocking phagosome acidification to resist the microbial killing action of phagocytic cells, and by releasing superoxide dismutase to resist the respiratory burst.^[10]^

*Nocardia* infections are commonly observed in immunosuppressed patients, with frequent underlying causes including malignancies, organ or hematopoietic stem cell transplantation, and human immunodeficiency virus infection. Other associated diseases include diabetes, chronic granulomatous disease, pulmonary alveolar proteinosis, inflammatory bowel disease, chronic obstructive pulmonary disease, and tuberculosis.^[11]^ In most cases, *Nocardia* infections cannot be rapidly diagnosed due to lack of specific clinical symptoms. The clinical manifestations of *Nocardia* infection, commonly referred to as primary *Nocardia* pneumonia, primarily include fever, cough with sputum, hemoptysis, chest pain, dyspnea, and anorexia, with some patients exhibiting no clinical symptoms.^[12]^ In this case, the patient presented with cough, sputum production, and a pulmonary mass, lacking other specific symptoms, and had suboptimal response to anti-inflammatory treatment, leading to consideration of neoplastic disease.

The chest imaging manifestations of PN are diverse, including consolidation, cavitary and non-cavitary pulmonary nodules/masses, ground glass opacities, centrilobular nodules, interlobular septal thickening, “crazy paving” pattern, pleural effusion, and pleural traction, with the most common imaging finding being nodules or masses. Additionally, lymphadenopathy is observed in some patients.^[13,14]^ Kurahara et al,^[15]^ in 2014 conducted an imaging analysis on 59 patients with PN, of which 28 showed airspace consolidation, 13 showed nodules, 3 showed consolidation with cavity, 2 showed cavity, 1 showed pleural effusion, and 12 showed radiographic patterns were indeterminate. The combination of these imaging findings with nonspecific clinical symptoms often leads to misdiagnosis as tuberculosis, fungal infections, or malignancies. In a meta-analysis,^[16]^ 4 studies with a total sample size of 476 suspected tuberculosis patients were included, revealing a prevalence of PN ranging from 1.7% to 6.7%. Chronic structural lung diseases and long-term use of immunosuppressive agents are risk factors for PN. Although CT manifestations are highly heterogeneous, the presence of nodules, patchy consolidation, and cavities, especially in the context of extrapulmonary infections such as brain and subcutaneous tissue, should raise clinical suspicion. The incidence of cavities is significantly higher in immunosuppressed patients.^[17]^ The 1-year mortality rate of nocardiosis ranges from 15.8% to 24.5%, varying according to the type of infecting organism and the patient’s immune status.^[18]^ Prognosis depends on the extent of the disease and complications, but early diagnosis and timely treatment can reduce mortality and recurrence risks.^[19]^ *Nocardia* can be isolated from respiratory secretions, blood, pus, or tissue samples through culture or molecular techniques, which are essential for the diagnosis of PN. Rapid molecular identification of *Nocardia* can be achieved through methods such as polymerase chain reaction, deoxyribonucleic acid probes, deoxyribonucleic acid sequencing, pyrosequencing, ribotyping, and restriction fragment length polymorphism analysis.^[5]^ Diagnosis requires clinical symptoms, as well as microbiological idenification of *Nocardia* species from clinical specimens such as sputum, bronchoalveolar lavage fluid, fine needle aspiration, or lung biopsy, *Nocardia amamiensis* was detected by 16S ribosomal ribonucleic acid sequencing of the sputum specimen are reported by Kanakan et al.^[4]^ 16S ribosomal ribonucleic acid sequencing as a simpler alternative to metagenomic NGS which may not be available. In this case, the diagnosis was confirmed by performing metagenomic NGS on the pathological tissue.

Since 1950, sulfonamides have remained the cornerstone of *Nocardia* infection treatment, with TMP-SMX most common.^[20]^ Several other drugs show activity against *Nocardia*, including carbapenems, ceftriaxone, amikacin, minocycline, fluoroquinolones, and amoxicillin-clavulanate. Linezolid and tedizolid expand treatment options and are active against most *Nocardia* species in vitro.^[21]^ Combination therapy is often used for prolonged disease course, severe or disseminated infections. Immunocompromised patients require longer treatment. CT consolidation and nodules indicate poorer prognosis, potentially progressive pulmonary *Nocardia* infection, and more aggressive treatment is recommended.^[22]^ The patient was initially treated for lung cancer and obstructive pneumonia with ineffective common bacterial antibiotics. After *Nocardia* infection diagnosis and TMP-SMX treatment, chest CT lesion absorption improved significantly.

PN is a rare opportunistic infectious disease. In immunocompetent patients, the diagnosis of *Nocardia* is particularly challenging due to the nonspecific nature of symptoms and imaging findings. Therefore, in patients without immunosuppression, especially those with frequent exacerbations of chronic obstructive pulmonary disease, if symptoms and imaging features suggestive of lung cancer are present, it is crucial to be vigilant for PN. To accurately diagnose diseases caused by rare bacterial species, repeated sampling and the identification of appropriate testing methods are paramount.

## Author contributions

**Data curation:** Qian Liu, Qian Li, Bing Liu, Tian Fu.

**Conceptualization:** Tian Fu.

**Investigation:** Qian Liu, Tian Fu.

**Supervision:** Bing Liu, Tian Fu.

**Writing – original draft:** Qian Liu, Tian Fu.

**Writing – review & editing:** Qian Liu, Tian Fu.

## Supplementary Material
