# Supplementary material for: Pulmonary nocardiosis with hilar mass misdiagnosed as lung cancer: A case report
Source: Medicine (Baltimore). 2025 May 16;104(20):e42524. doi: 10.1097/MD.0000000000042524 (PMC12091657; doi:10.1097/MD.0000000000042524)
Supplement: Supplementary file 1 [file medi-104-e42524-s001.pdf]

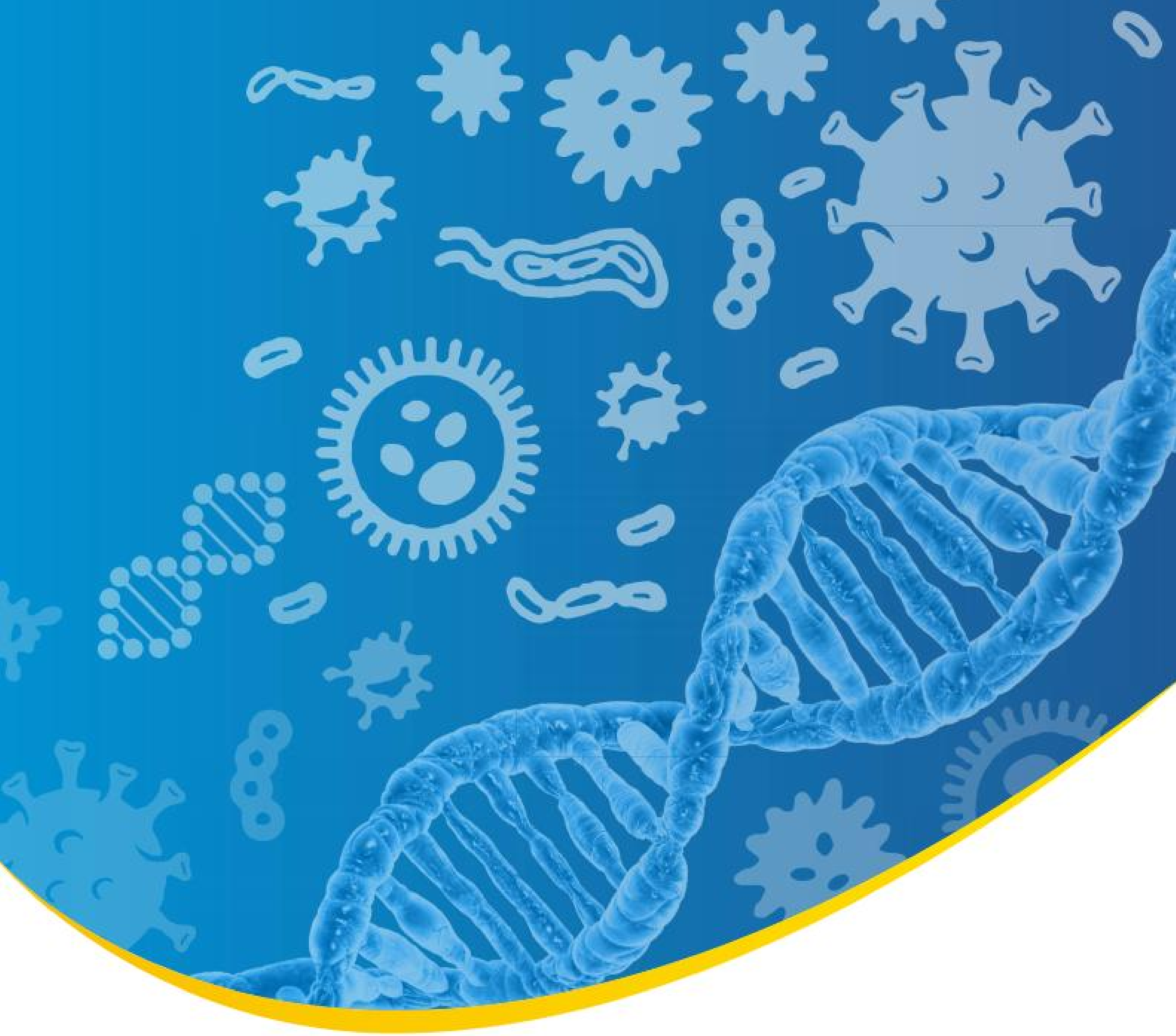

病原微生物宏基因组报告

姓　　名：[redacted]

样本编号：A41001005585

广州华银医学检验中心

一、样本信息

受检者信息

姓名：[redacted]	性别：女
电话：-	年龄：54 岁
住院号：[redacted]	床号：-

送检方信息

单位：济宁市第一人民医院	
科室：呼吸与危重症西院病区	医生：刘冰

样品信息

样品编号：A41001005585	样品类型：蜡卷
采样日期：2023-05-05	送检日期：2023-05-05
检测日期：2023-05-06	报告日期：2023-05-08

临床信息

主诉：-	
临床诊断：-	
是否使用过抗生素：-	发病时长：
白细胞计数：-	中性粒细胞比率：-
淋巴细胞比率：-	降钙素原（PCT）：-
C 反应蛋白（CRP）：-	培养鉴定结果：-

检测项目

J101502 宏基因组学检测（细菌，真菌，寄生虫，DNA 病毒）

二. 检测结果

DNA 流程检测结果：疑似阳性

1. 细菌

皮疽诺卡菌(607)，检出人体常见定植菌（具体见疑似微生态列表）

2. 真菌

未检出疑似病原体，检出人体常见定植菌（具体见疑似微生态列表）

3. 病毒

未检出疑似病原体

4. 寄生虫

未检出疑似病原体

5. 特殊病原体（包括分枝杆菌、支原体/衣原体）

未检出

6. 耐药基因

未检出

7. 毒力基因

检测者：[redacted] 审核者：[redacted] 报告日期：2023-05-08

GUANGZHOU HUAYIN MEDICAL LABORATORY CENTER 广州华银医学检验中心 报告专用章 (一)

三. 详细检测结果

1. 细菌					
属				种	
革兰氏染色	属名	相对丰度(%)	序列数	种名	序列数
G+	诺卡氏菌 *Nocardia*	20.57	696	皮疽诺卡菌 *Nocardia farcinica*	607

2. 真菌
未发现

3. DNA 病毒
未发现

4. RNA 病毒
未进行 RNA 流程检测

5. 寄生虫
未发现

6. 结核分枝杆菌复合群
未发现

7. 非结核分枝杆菌
未发现

8. 支原体/衣原体
未发现

病原体解释

1) **皮疽诺卡菌(*Nocardia farcinica*)：**

革兰阳性杆菌，诺卡菌属。多引起外源性感染，有毒株为兼性胞内寄生菌。在自然界分布广泛，多为腐生寄生菌，是一种机会致病菌。目前国内关于该菌引起感染的致病报道罕见。

覆盖到基因组上的总长度为 33597 (bp),覆盖度为 0.5225% ,平均深度为 1.01 X。

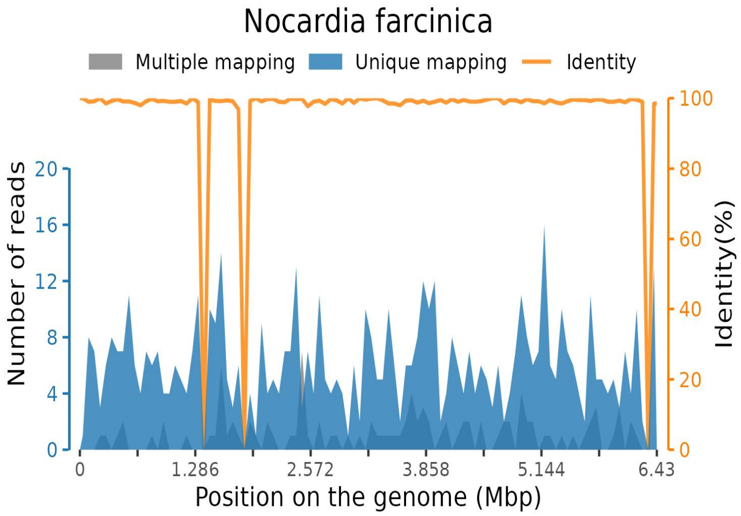

9. 疑似人体微生态菌群					
属				种	
类型	属名	相对丰度	序列数	种名	序列数
G+	丙酸杆菌属 *Cutibacterium*	19.95	675	痤疮丙酸杆菌 *Cutibacterium acnes*	541
G+	棒杆菌属 *Corynebacterium*	3.55	120	变异棒状杆菌 *Corynebacterium variabile*	25
G+	葡萄球菌属 *Staphylococcus*	3.28	111	表皮葡萄球菌 *Staphylococcus epidermidis*	43
G+	葡萄球菌属 *Staphylococcus*	3.28	111	沃氏葡萄球菌 *Staphylococcus warneri*	32
G+	葡萄球菌属 *Staphylococcus*	3.28	111	人葡萄球菌 *Staphylococcus hominis*	14
G-	莫拉菌属	2.42	82	奥斯陆莫拉菌	80

	Moraxella			*Moraxella osloensis*	
G+	微球菌属<br>*Micrococcus*	1.92	65	藤黄微球菌<br>*Micrococcus luteus*	32
fun	念珠菌属<br>*Candida*	2.50	5	近平滑念珠菌<br>*Candida parapsilosis*	5

微生态菌群解释

1) 痤疮丙酸杆菌(*Cutibacterium acnes*)：

革兰阳性杆菌，丙酸杆菌属。痤疮丙酸杆菌是皮肤上的优势菌群，栖居于毛囊、皮脂腺内，可从人的鼻腔、口腔、肠道和泌尿道中分离。此外，该菌是植入修复物或器械引起感染的主要病原菌，对原有心瓣膜损伤者可引起心内膜炎，是感染性心内膜炎的罕见病因。国外有报道该菌可引起中枢神经系统感染、眼部感染、口腔感染及呼吸系统感染。

2) 变异棒状杆菌(*Corynebacterium variabile*)：

革兰阳性杆菌，棒状杆菌属。棒状杆菌可分离自土壤、水体、血液及人类皮肤，变异棒状杆菌被认为是非致病性棒状杆菌，目前无感染人类的相关报道。

3) 表皮葡萄球菌(*Staphylococcus epidermidis*)：

凝固酶阴性革兰阳性球菌，葡萄球菌属。表皮葡萄球菌是人体皮肤和黏膜上定居的正常菌群之一，为人类机会致病菌，通常情况下致病力很低。近几年来，随着留置静脉导管等侵袭性操作的增多，该菌可寄居在医疗设备上（如人工心脏瓣膜），引起术后感染及亚急性细菌性心内膜炎、腹膜炎、泌尿系感染、眼内炎、中耳炎及各类伤口感染等，已成为医院感染的重要致病菌。表皮葡萄球菌感染的诊断至今仍是一个难题，由于从实验的各个环节都有可能被污染，一般必须进行多次培养同时检出并结合临床才能作出诊断。

4) 沃氏葡萄球菌(*Staphylococcus warneri*)：

革兰氏阳性的球形细菌，葡萄球菌属。主要存在于环境及人与动物的皮肤和粘膜上，有报道认为沃氏葡萄球菌与人类的动脉栓塞和牙周炎存在一定的关系。

5) 人葡萄球菌(*Staphylococcus hominis*)：

凝固酶阴性革兰阳性球菌，葡萄球菌属。属于人类皮肤正常菌群。可引起免疫功能低下患者的机会性感染，使其患有败血症、心内膜炎等，是一种少见的条件致病菌，主要定植于人体皮肤表面和黏膜表面。

6) 奥斯陆莫拉菌(*Moraxella osloensis*)：

革兰氏阴性球杆菌，莫拉菌属。是人和动物粘膜的正常菌群，偶见于菌血症、心内膜炎、脑膜炎、肺炎、关节炎等感染。

7) 藤黄微球菌(*Micrococcus luteus*):

革兰阳性球菌，微球菌属。主要存在于泥土、水等外界环境以及人和动物的皮肤表面。一般不致病，在自然界和临床标本中较为常见，需要根据标本来源、菌落数量、菌落是否生长在接种线上等因素综合判断区分定植菌、污染菌还是感染菌。当人体免疫力低下时可引起各种机会感染，如菌血症、脑膜炎、心内膜炎等。

8) 近平滑念珠菌(*Candida parapsilosis*):

念珠菌属，呈酵母样。近平滑念珠菌是一种常见的非白念珠菌，致病力较低，通常可以在皮肤、黏膜、外耳道等部位分离出，临床常难以区分侵袭性感染与无症状定植，患者临床表现可从局部粘膜病变（局部过度增殖和侵袭性感染）到播散性感染（血源性感染）。在自然界分布广泛，可存在于健康人的黏膜表面、皮肤以及指甲中。身体皮肤表面是最主要的分离部位，可引起皮肤浅表感染、中耳炎、鼻窦炎、眼和泌尿生殖道感染。从感染人群来看，近平滑念珠菌是低体重新生儿最常见的血流感染病原体。近平滑念珠菌、拟平滑念珠菌、似平滑念珠菌合称为近平滑念珠菌复合群。

10. 耐药基因
未发现

注：[a] 耐药基因与表型之间存在差异，检出耐药基因并不能确认该菌对相应药物一定耐药，本检测结果仅供临床参考，请以临床医生指导用药为准。

[b] 更多耐药基因相关信息详见于：https://card.mcmaster.ca/

耐药基因解释

注：病原体解释参考来自《ABX 指南》、《临床微生物学手册（第 11 版）》等书籍。

名词解释

相对丰度：将病原体按照细菌、真菌、病毒和寄生虫进行分类，相对丰度是该病原体相应分类中基因组相对比例。

序列数：高通量测序序列唯一比对到某属或种微生物特异性序列数。

人体微生态菌群：存在于人体皮肤、呼吸道、口腔、胃肠道、泌尿道的微生物，多为条件致病菌，正常条件下与人体共生，在免疫力低下/缺陷的患者中具有潜在致病性。

覆盖度图：针对特定微生物绘制图谱，反映比对到该微生物的序列在其基因组上的分布情况，横坐标代表该微生物的基因组大小，纵坐标代表不同基因组区段内检出的序列数。

四．检测质控

实验质控	
质控参数	样本数据
内参	合格
核酸提取浓度（ng/μL）	60.95
文库浓度（ng/μL）	13.10
阴控	合格
是否去宿主	否

数据质控		
总数据量	非人源序列数	Q30 比率（%）
55184616	3545616	94.06

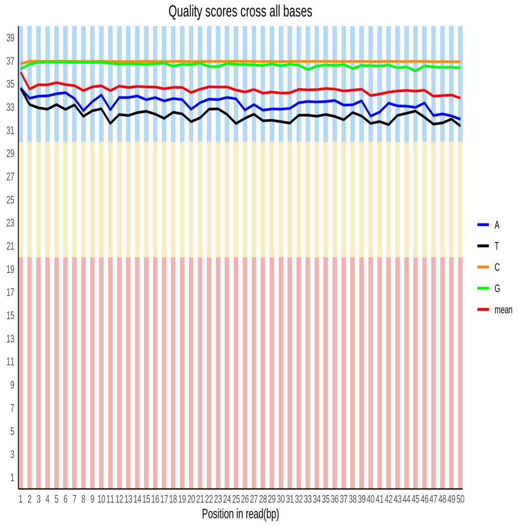

注：以上图表说明此次检测测序数据质量合格，结果可信。

五. 检测流程和范围

检测流程

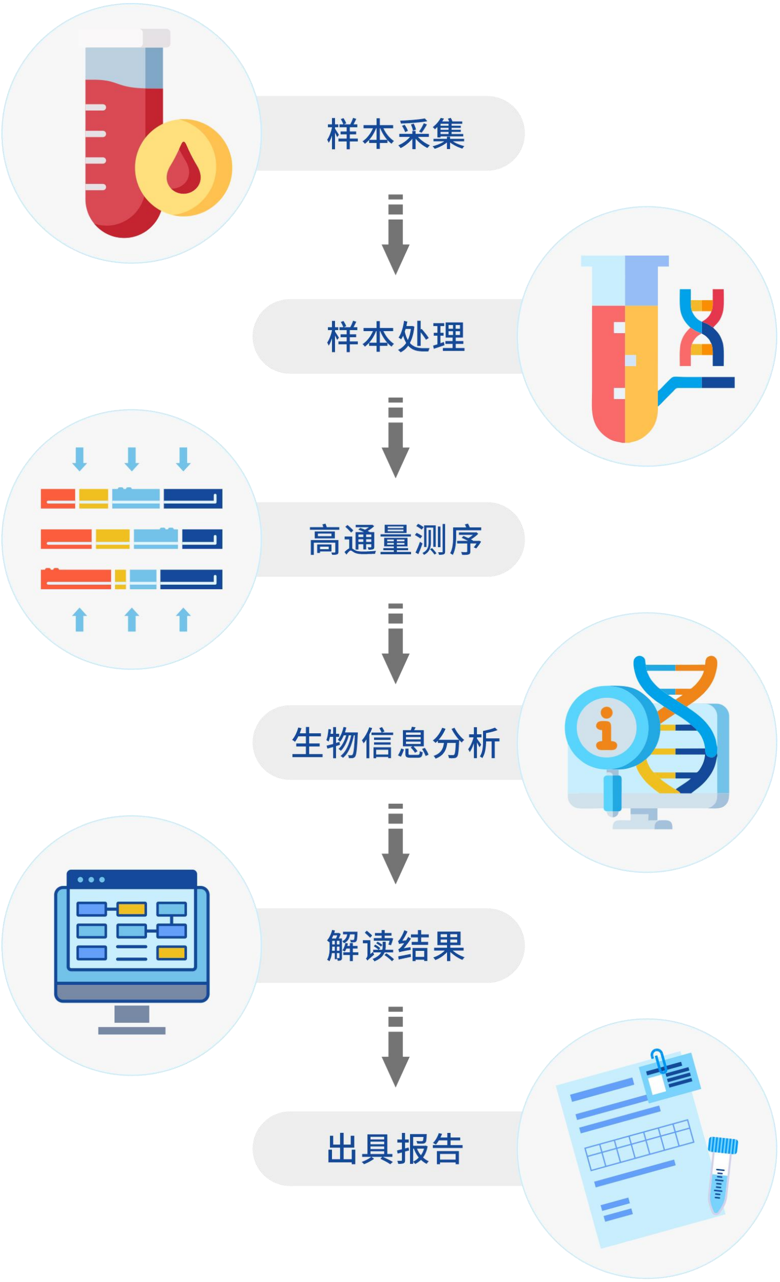

检测范围

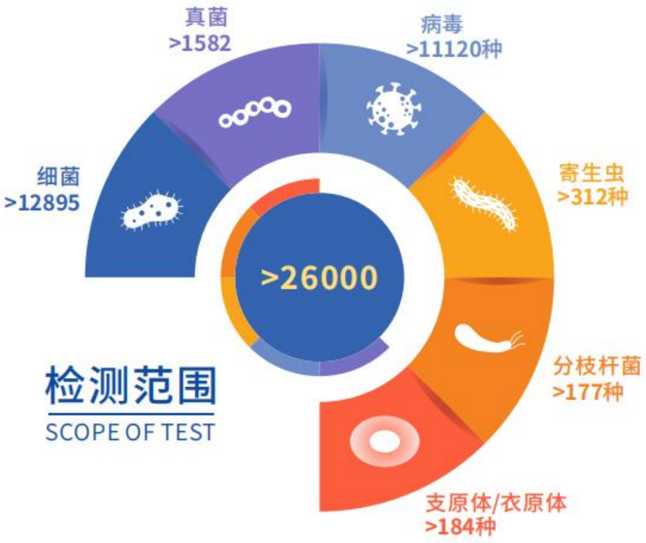

人体微生态菌群

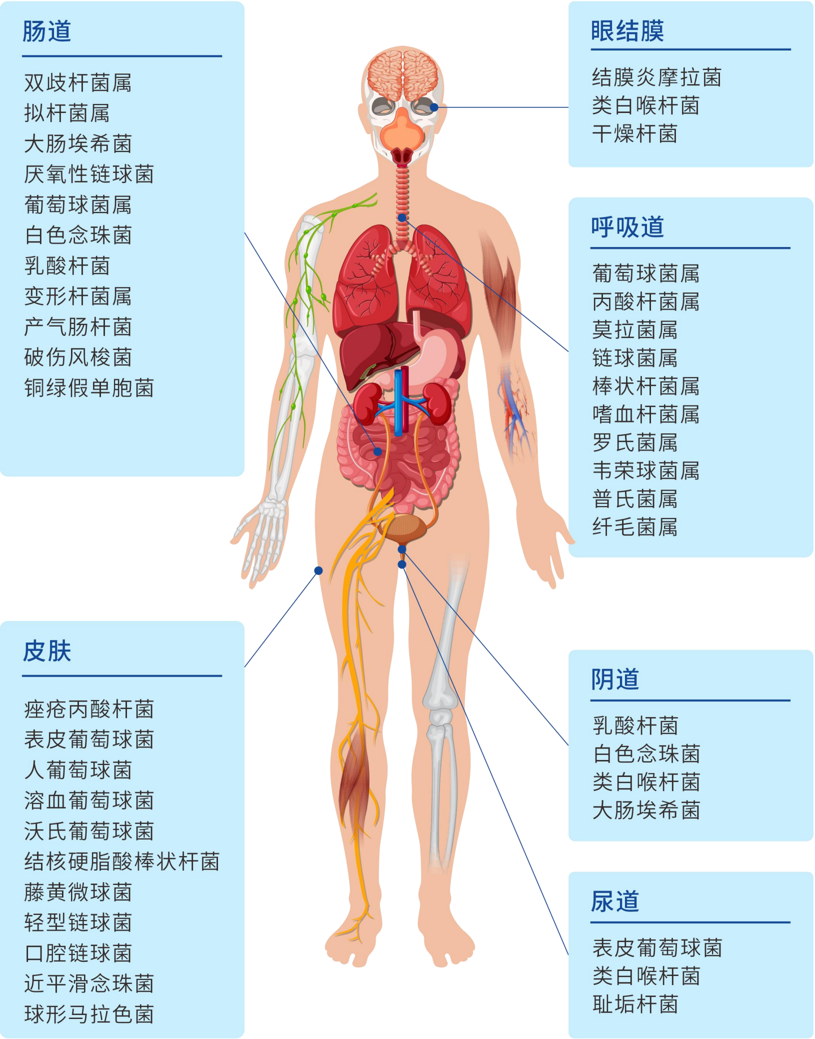

六. 检测方法学及局限性

病原宏基因组学与宏转录组学检测方法是一种新的不依赖传统微生物培养而广泛分析临床样本中微生物组(细菌、真菌、病毒和寄生虫)的高通量测序方法[1]。该技术通过高通量测序和智能化算法分析，可以获得疑似致病微生物的种属信息，无偏向性鉴定细菌、真菌、病毒和寄生虫等多种病原微生物[2]。适用于不明原因发热、疑难危重以及免疫缺陷感染患者[3-5]，目前已有多篇用于脓毒症、脑膜炎、呼吸系统感染等方面的报道[6-8]。

1. 本检测非临床常规检测项目，仅供临床参考，临床相关解释须咨询临床医生，如有其它疑义请在七个工作日内与我们联系。
2. 样本采集，保存，运输等环节的不当操作可能影响本产品检测性能。
3. 本检测结果仅对该次送检样本负责。受方法学检测限影响，微生物载量过低、当前数据库构建局限或用药等因素可能使得检测结果未涵盖样本中所有的微生物。
4. 检测方将严格依法保护患者隐私与检测结果。

七. 病原宏基因组学参考文献

[1] Wilson,M.R.,et al.,Actionable diagnosis of neuroleptospirosis by next-generation sequencing. N Engl J M ed,2014.370(25): p.2408-17.

[2] Dekker,J.P.,Metagenomics for Clinical Infectious Disease Diagnostics Steps Closer to Reality. J Clin Microbiol,2018.56(9).

[3] Thoendel,M .J.,et al.,Identification of Prosthetic Joint Infection Pathogens Using a Shotgun Metagenomics Approach. Clin Infect D is,2018.67(9): p.1333-1338.

[4] Brown,J.R .,T.B harucha,and J.B reuer,Encephalitis diagnosis using metagenomics: application of next generation sequencing for undiagnosed cases. J Infect,2018.76(3): p.225-240.

[5] Parize,P.,et al.,Untargeted next-generation sequencing-based first-line diagnosis of infection in immunocompromised adults: a multicentre, blinded, prospective study. Clin Microbiol Infect,2017.23(8): p.574 e1-574 e6.

[6] Blauwkamp,T.A.,et al.,Analytical and clinical validation of a microbial cell-free DNA sequencing test for infectious disease. Nat Microbiol,2019,4(4),p.663.

[7] Miller,S.,et al.Laboratory validation of a clinical metagenomic sequencing assay for pathogen detection in cerebrospinal fluid. Genome Res,2019.

[8] Langelier,C .,et al.Integrating host response and unbiased microbe detection for lower respiratory tract infection diagnosis in critically ill adults. Proc Natl Acad Sci USA, 2018,115(52):E12353-E12362.

[9] 《中华传染病杂志》编辑委员会. 中国宏基因组学第二代测序技术检测感染病原体的临床应用专家共识. 中华传染病杂志,2020,38 (11): 681-689.

广州华银医学检验中心
Guangzhou Huayin Medical Laboratory Center

广州市科学城揽月路 80 号广州科技创新基地

No.80 Lanyue Rd.Guangzhou science and InnovationBase,GZ.

华银康官微
